# Promoted ABA Hydroxylation by *Capsicum annuum CYP707As* Overexpression Suppresses Pollen Maturation in *Nicotiana tabacum*

**DOI:** 10.3389/fpls.2020.583767

**Published:** 2020-12-08

**Authors:** Hyun Min Kim, Se Hee Park, Sang Hoon Ma, Seo Young Park, Chul-Ho Yun, Geupil Jang, Young Hee Joung

**Affiliations:** School of Biological Sciences and Technology, Chonnam National University, Gwangju, South Korea

**Keywords:** ABA hydroxylation, CaCYP707A, *Capsicum annuum*, cytochrome P450, pollen maturation, pollen viability, seed formation

## Abstract

Abscisic acid (ABA) is a key signaling molecule that mediates plant response to stress. Increasing evidence indicates that ABA also regulates many aspects of plant development, such as seed germination, leaf development, and ripening. ABA metabolism, including ABA biosynthesis and degradation, is an essential aspect of ABA response in plants. In this study, we identified four cytochrome P450 genes (*CaCYP707A1*, *2*, *3*, and *4*) that mediate ABA hydroxylation, which is required for ABA degradation in *Capsicum annuum*. We observed that CaCYP707A-mediated ABA hydroxylation promotes ABA degradation, leading to low levels of ABA and a dehydration phenotype in *35S:CaCYP707A* plants. Importantly, seed formation was strongly inhibited in *35S:CaCYP707A* plants, and a cross-pollination test suggested that the defect in seed formation is caused by improper pollen development. Phenotypic analysis showed that pollen maturation is suppressed in *35S:CaCYP707A1* plants. Consequently, most *35S:CaCYP707A1* pollen grains degenerated, unlike non-transgenic (NT) pollen, which developed into mature pollen grains. Together our results indicate that CaCYP707A mediates ABA hydroxylation and thereby influences pollen development, helping to elucidate the mechanism underlying ABA-regulated pollen development.

## Introduction

Plant hormones regulate plant physiology and growth by governing endogenous developmental programs and plant responses to environmental signals. Abscisic acid (ABA) is an essential hormone that mediates plant defense against environmental stresses (Sah et al., 2016). For example, plants with reduced ABA response show reduced tolerance to abiotic stresses (drought, salinity, and oxidative stresses), whereas plants with enhanced ABA response exhibit improved tolerance (Verslues and Bray, 2006; Dong et al., 2011; Vishwakarma et al., 2017). In addition, increasing evidence shows that ABA is deeply involved in aspects of plant development such as germination, stomate formation, and development of roots, leaves, and fruit (Nakashima and Yamaguchi-Shinozaki, 2013; Tanaka et al., 2013; Leng et al., 2014; Chater et al., 2015; Steinbrecher and Leubner-Metzger, 2017; Yoshida et al., 2019).

The ABA signaling pathway comprises several signaling components, including PYRABACTIN RESISTANCE 1 (PYR1)/PYR-LIKE (PYL)/REGULATORY COMPONENT OF ABA RECEPTOR (RCAR), PROTEIN PHOSPHATASE TYPE 2C (PP2C), and SNF1-RELATED PROTEIN KINASE TYPE 2 (SnRK2) (Ma et al., 2009; Park et al., 2009; Umezawa et al., 2009; Vlad et al., 2010). Mutant plants with defects in expression of *PYL* and *SnRK2* genes show suppressed ABA responses (Fujii et al., 2011; Gonzalez-Guzman et al., 2012), indicating that ABA signaling is critical for ABA responses in plants. Briefly, ABA signaling is initiated by perception of ABA by the PYR1/PYL/RCAR family of signaling components (Ma et al., 2009; Park et al., 2009). PYR–ABA interaction suppresses the activity of PP2C proteins, which are responsible for the repression of downstream kinases such as SnRK2s, which themselves are positive regulators of ABA signaling (Cutler et al., 2010; Antoni et al., 2012; Soon et al., 2012). The kinase activities of SnRK2s activate ABA-RESPONSIVE ELEMENT BINDING FACTORS, leading to expression of ABA-responsive genes and promoting the ABA response (Furihata et al., 2006; Finkelstein, 2013; Ng et al., 2014).

The ABA response is also regulated by ABA metabolism, which includes biosynthesis, conjugation, and degradation. ABA biosynthesis involves the production of the ABA precursor xanthoxin in plastids and the conversion of xanthoxin to ABA in the cytoplasm (Seo and Koshiba, 2002; Seiler et al., 2011). Xanthoxin production, carried out by 9-*cis-*epoxycarotenoid dioxygenase (NCED), is a key step in ABA biosynthesis (Iuchi et al., 2001; Schwartz et al., 2003; Tan et al., 2003). Mutant plants that lack NCED activity show suppressed ABA accumulation and responses, whereas plants that overexpress NCED exhibit enhanced ABA responses (Tan et al., 1997; Burbidge et al., 1999; Iuchi et al., 2001; Lefebvre et al., 2006; Rodrigo et al., 2006; Zhang et al., 2009; Sun et al., 2012; Priya and Siva, 2015). Free ABA can be further metabolized by UDP glucosyltransferase into an ABA–glucose ester through conjugation with glucose (Dietz et al., 2000; Han et al., 2019).

Degradation is another key process controlling cellular ABA levels. Although ABA–glucose ester conjugation is involved in ABA degradation, it has been suggested that the main process by which ABA is degraded is ABA hydroxylation, which is mediated by several cytochrome P450 (CYP) monooxygenases (CYP707As) (Krochko et al., 1998; Dong and Hwang, 2014; Han et al., 2019). The activity of CYP707As produces 8′-hydroxy ABA, which spontaneously converts into phaseic acid (PA) and is further metabolized into dihydrophaseic acid by PA reductase (Kushiro et al., 2004; Saito et al., 2004; Nambara and Marion-Poll, 2005; Rodriguez, 2016; Weng et al., 2016). CYP707As with ABA 8′-hydroxylase activity were conserved during land plant evolution (Zheng et al., 2012), and there are three CYP707As in rice, four in Arabidopsis, and seven in papaya (Kushiro et al., 2004; Saika et al., 2007; Nelson et al., 2008). *CYP707A* genes show tissue-specific expression patterns that change with the developmental stage (Okamoto et al., 2006; Nitsch et al., 2009; Wang et al., 2013; Brun et al., 2019). In addition, similar to other genes involved in ABA signaling and biosynthesis, expression levels of *CYP707As* are regulated by abiotic stresses such as drought, salinity, and oxidative stress (Yang and Zeevaart, 2006; Okamoto et al., 2009; Ren et al., 2010; Zheng et al., 2012; Wang et al., 2013; Ji et al., 2014). Furthermore, knockout or knockdown of *CYP707As* promotes seed dormancy and fruit ripening by increasing ABA levels (Kushiro et al., 2004; Okamoto et al., 2009; Ji et al., 2014), and inactivation of CYP707As with a chemical inhibitor mimics the effect of *CYP707A* mutation (Suttle et al., 2012), supporting that *CYP707As* play an essential role in regulation of ABA metabolism and response.

Hot pepper (*Capsicum annuum*) is a widely cultivated crop around the world (Pathirana, 2013), and the fruit productivity and quality are sensitive to environmental stresses (Guang-Cheng et al., 2010; Navarro et al., 2010; Unlukara et al., 2013). Because of this sensitivity, the role of ABA in stress responses in *C. annuum* has been widely studied (Lee and Choi, 2013; Guan et al., 2018; Hou et al., 2018; Jin et al., 2019; Ruggiero et al., 2019; Xiao et al., 2020). In this study, we attempted to isolate CYPs mediating ABA hydroxylation in *C. annuum* and identified four: CaCYP707A1, 2, 3, and 4. Enzymatic assays showed that the CYP707As have ABA hydroxylation activity, and expression analysis indicated that *CaCYP707A* transcription is regulated by abiotic stresses such as drought. In addition, transgenic tobacco overexpressing *CaCYP707A* genes (*35S:CaCYP707As*) exhibited a dehydration phenotype under normal growth conditions, unlike non-transgenic (NT) control plants, suggesting that *CaCYP707A* genes are responsible for ABA metabolism and responses. Moreover, overexpression of CaCYP707As suppressed seed formation, and analysis of pollen viability and ultrastructure showed that the suppression of seed formation was caused by defects in pollen maturation. These findings suggest that CaCYP707As-mediated ABA hydroxylation is involved in pollen maturation and seed formation.

## Materials and Methods

### Plant Materials and Growth Conditions

*Nicotiana tabacum* cv. Xanthi-nc was used to generate *35S:CaCYP707A* and NT control plants. *Capsicum annuum* L. cv. Bukang was used for isolation of *CaCYP707As* and for characterization of *CaCYP707A* expression in response to drought. All plants were grown at 22 ± 1°C with a controlled light (120 μmol m^–2^ s^–1^) cycle (16 h light/8 h dark).

### Isolation of *CYP707As* and Multiple Alignment

To isolate *CYP707As*, total RNA extraction and cDNA biosynthesis from *C. annuum* leaves and flowers were carried out as described previously (Kim et al., 2019). The full-length *CaCYP707A* cDNAs were amplified by PCR from the *C. annuum* cDNA and cloned into pGEM-T Easy Vectors (Promega, United States). The inserted sites were sequenced and aligned with their homologs from Arabidopsis and tomato using the PRALINE toolbox (Simossis and Heringa, 2005). CaCYP707A protein sequences were deduced from the nucleic acid sequences and uploaded to GenBank with the following accession numbers: CaCYP707A1 (MT198680), CaCYP707A2 (JQ828939), CaCYP707A3 (MT198681), and CaCYP707A4 (MT198682). Primer information for isolation of *CaCYP707A* genes is listed in Supplementary Table 1.

### Subcellular Localization of CaCYP707As

Subcellular localization analysis was carried out as previously described (Voinnet et al., 2003; Kim et al., 2016), with a slight modification. 35S:*CaCYP707A*-*GFP* plasmids were constructed by inserting full-length of *CaCYP707A* cDNAs into a pBI121-GFP plasmid carrying the *35S* promoter and the *GFP* gene; these were inserted into tobacco leaves (*Nicotiana benthamiana*) by agro-infiltration. To verify the subcellular localization of CaCYP707A-GFP, endoplasmic reticulum (ER)-targeting mCherry protein (ER-mCherry) and plasma membrane (PM)-targeting mCherry protein (PM-mCherry) plasmids were used (Nelson et al., 2007); these were co-introduced into tobacco leaves together with the 35S:*CaCYP707A*-GFP plasmids. After 5 days, GFP and mCherry fluorescence signals were observed with a laser-scanning confocal microscope (Leica TCS SP5, Germany). Primer information for construction of 35S:*CaCYP707A*-*GFP* is listed in Supplementary Table 1.

### Analysis of ABA Hydroxylation Activity of CaCYP707As

Full-length *CaCYP707A* cDNAs were ligated to pCW vectors and introduced into the *Escherichia coli* strain Rosetta (DE3) pLysS. The expression and purification of CaCYP707As were conducted as previously described (Kim et al., 2008). The concentrations of isolated CaCYP707A proteins were determined using the co-difference spectra method (Omura and Sato, 1964). The isolated CaCYP707A proteins were incubated with substrate (0.1 mM ABA), reductase, and a NADPH-generating system (NGS) in 0.1 M KPi buffer (pH 7.4). As the reductase, CaCPR1, which was confirmed to have reductase activity in a previous study (Lee et al., 2014), was used. The NGS contained 10 mM glucose-6-phosphate, 0.5 mM NADP^+^, and 1 IU/ml glucose 6-phosphate dehydrogenase (Spaans et al., 2015). The negative controls were conducted without NGS solution. After a 30-min incubation at 27°C, the reaction was terminated with 25 μl of 1 N HCl. The reaction products were collected by addition of ethyl acetate (three times) and dried using a stream of nitrogen gas. Analysis of product formation was conducted using an HPLC system (Shimadzu, Japan) with a UV detector and a Gemini C18 column (4.6 mm × 150 mm, 5 μm, Phenomenex). The mobile phase solution was 0.05% acetic acid and 45% methanol in water (v/v). The flow rate was 1 ml/min, and detection was conducted at 254 nm. Primer information for insertion into pCW vectors is listed in Supplementary Table 1.

### Generation of *35S:CaCYP707A* Transgenic Plants

To construct *35S:CaCYP707A* genes, full-length *CaCYP707A* cDNAs were inserted into the pBI121 binary vector with the 35S promoter for overexpression of *CaCYP707As*. The recombinant plasmids were introduced into tobacco using *Agrobacterium tumefaciens strain* LBA4404–mediated transformation as described previously (Oh et al., 2005), with a slight modification. For transgenic plant generation, tobacco leaves (*N. tabacum* cv. Xanthi-nc) were cut into small disks and incubated with *A. tumefaciens* for 10 min, after which the explants were placed on co-culture medium. After 2 days, explants were transferred to shoot induction medium [Murashige and Skoog medium containing 3% (w/v) sucrose, 1 μg/ml zeatin, 0.01 μg/ml 1-naphthaleneacetic acid, 0.1 μg/ml gibberellic acid, 50 μg/ml kanamycin, and 250 μg/ml cefotaxime, pH 5.8] and then transferred to new medium every 2 weeks. After 6–8 weeks, the developing shoots were excised from the callus and transferred to rooting medium [Murashige and Skoog medium containing 1.5% (w/v) sucrose, 2 μg/ml 1-naphthaleneacetic acid, 50 μg/ml kanamycin, and 250 μg/ml cefotaxime, pH 5.8]. After the root induced, these regenerated plants were transferred to soil. Primer information for construction of 35S:*CaCYP707A* genes is listed in Supplementary Table 1.

### Phenotypic Analysis of Pollen Development

For the analysis of pollen ultrastructure, anthers were collected from tobacco flowers at early and middle developmental stages and then were fixed and observed as described previously (Lee et al., 2017), with minor modifications. In brief, the anthers were fixed in 0.05 M sodium cacodylate buffer (pH 7.2) containing 2% (v/v) glutaraldehyde and 2% (w/v) paraformaldehyde and postfixed with 1% (w/v) osmium tetroxide (Sigma-Aldrich, United States) in the same buffer. After a series of ethanol dehydrations, each sample was embedded in LR White resin (Sigma-Aldrich, United States). For light-microscopic observation, semi-thin section samples were cut with a diamond knife at 2-μm increments in an ultramicrotome (RMC MT-X, United States) and stained with 0.5% (w/v) toluidine blue O (Sigma-Aldrich, United States). These samples were visualized using an Axio Lab A1 light microscope (Zeiss, Germany). For transmission electron microscopy (TEM), ultra-thin (80- to 100-nm) section samples were sliced using the instruments named above and collected on a nickel grid (carbon-film coated, 150 mesh). These samples were stained with 4% (w/v) uranyl acetate and 0.4% (w/v) lead citrate. Images of the ultra-thin section samples were obtained using a JEM-2100F microscope/camera (Jeol Ltd., Japan). For phenotypical analysis of pollen by scanning electron microscopy (SEM), pollen at the anthesis stage of flowering were collected with carbon sticky tape and coated with platinum. The images of pollen were captured with a JSM-IT300 microscope/camera (Jeol Ltd., Japan).

### Pollen Viability Assay

To test pollen viability, the pollen collected from flowers at the anthesis stage were incubated in staining solutions containing 12.5 μg/ml fluorescein diacetate (FDA) and 5 μg/ml propidium iodide (PI) for 5 min. Fluorescent signals (green signals for FDA; red for PI) were observed by fluorescence microscopy (Leica DM LB2, Germany).

### Quantitative PCR Analysis

*Capsicum annuum* leaves were detached from 5-week-old plants and then air-dried (drought-stressed) for 6 h in the dark condition. Total RNA was extracted from these samples, and cDNA was biosynthesized as described previously (Kim et al., 2019). To analyze changes of *CaCYP707A* expression in response to drought, quantitative PCR (qPCR) analysis was conducted with using a Rotor-Gene 6000 real-time amplification system (Corbett Research, Australia). The reaction mix consisted of cDNA, gene-specific primers, and QuantiTect SYBR Green Master Mix (Qiagen, Germany). All qPCR experiments were run three times in three biological replicates. The relative expression of the target gene for each repeated experiment was calculated by the 2^–ΔΔCt^ method as described previously (Livak and Schmittgen, 2001). Primer information for qPCR is listed in Supplementary Table 1.

### Quantification of ABA Concentration

Abscisic acid concentrations were measured as previously described (Han et al., 2012), with slight modifications. To analyze ABA concentrations in leaves and anthers, 100 mg samples of leaves were collected from 6-week-old tobacco plants and 100 mg of anthers were collected from the flowers at the anthesis stage, respectively. The collected samples were suspended in 1.5 ml of ABA extraction solution [0.45 mM butylated hydroxytoluene and 2.5 mM citric acid monohydrate in 80% (v/v) methanol] and incubated overnight at 4°C. Supernatants were collected and dried under vacuum conditions. Extracted ABA amounts were measured using an ABA ELISA quantitation kit (Agrisera, Sweden) according to the manufacturer’s instructions.

## Results

### Identification of CYP707As in *C. annuum*

**FIGURE 1:**
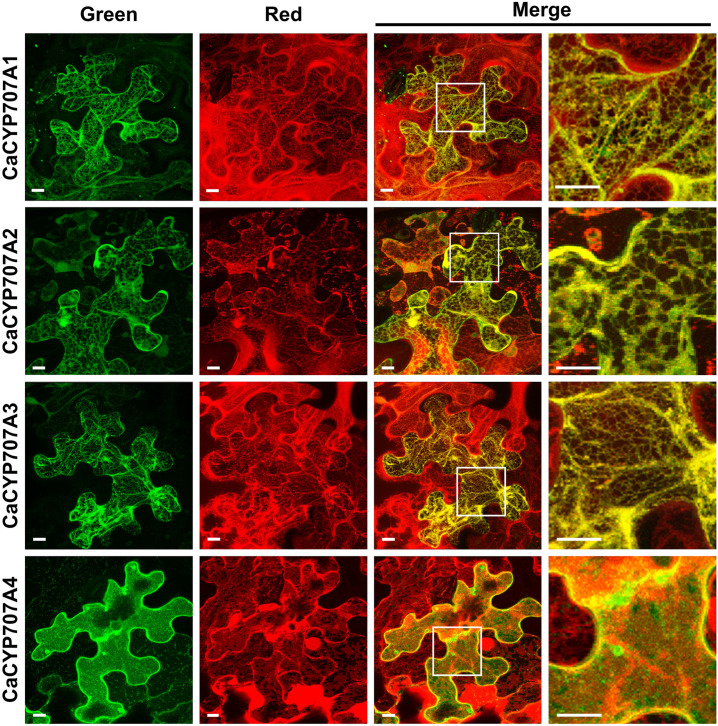
Subcellular localization of CaCYP707As. Subcellular localization of CaCYP707A1, 2, 3, and 4 proteins fused to GFP was investigated by visualizing fluorescent signals. ER-specific localization of CaCYP707A1, 2, and 3 proteins and PM-specific localization of CaCYP707A4 was verified through co-localization of ER-targeting mCherry (ER-mCherry) and PM-targeting mCherry (PM-mCherry), respectively. CYP707A1, 2, 3, and 4 indicate tobacco transiently expressing *35S:CaCYP707A1-GFP*, *2-GFP*, *3-GFP*, *and 4-GFP*. Green and red indicate fluorescence signals of GFP and mCherry, respectively. Scale bars = 10 μm. More than three individuals of the indicated plants were analyzed, and the experiments were performed three times with similar results.

Abscisic acid hydroxylation, which is involved in ABA degradation, regulates the ABA response in plants (Nambara et al., 2010; Ma et al., 2018). CYP707As mediate ABA hydroxylation (Krochko et al., 1998). Through bioinformatics approaches, we identified four *CYP707A* genes in the *C. annuum* genome, similar to the numbers in Arabidopsis and tomato (Kushiro et al., 2004; Nitsch et al., 2009). The four predicted *C. annuum* CaCYP707A proteins (CaCYP707A1, 2, 3, and 4) are highly conserved, as are those of Arabidopsis and tomato (Supplementary Figure 1). These findings suggest that the *CaCYP707A* genes encode CYP proteins that contribute to ABA hydroxylation. Because ABA is hydroxylated in ER by the activity of ER-localized CYP707As (Seo, 2014), we investigated subcellular localization of CaCYP707A1, 2, 3, and 4 by visualizing localization of GFP-fused CaCYP707A1, 2, 3, and 4 proteins. CaCYP707A1-GFP, CaCYP707A2-GFP, and CaCYP707A3-GFP signals were detected in the ER, where they co-localized with an ER marker. These findings suggest that the *CaCYP707A1*, *2*, and *3* genes encode CYPs that are responsible for ABA hydroxylation in the ER. By contrast, CaCYP707A4 localized to the plasma membrane (Figure 1).

### ABA Hydroxylation Activity of CaCYP707As

**FIGURE 2:**
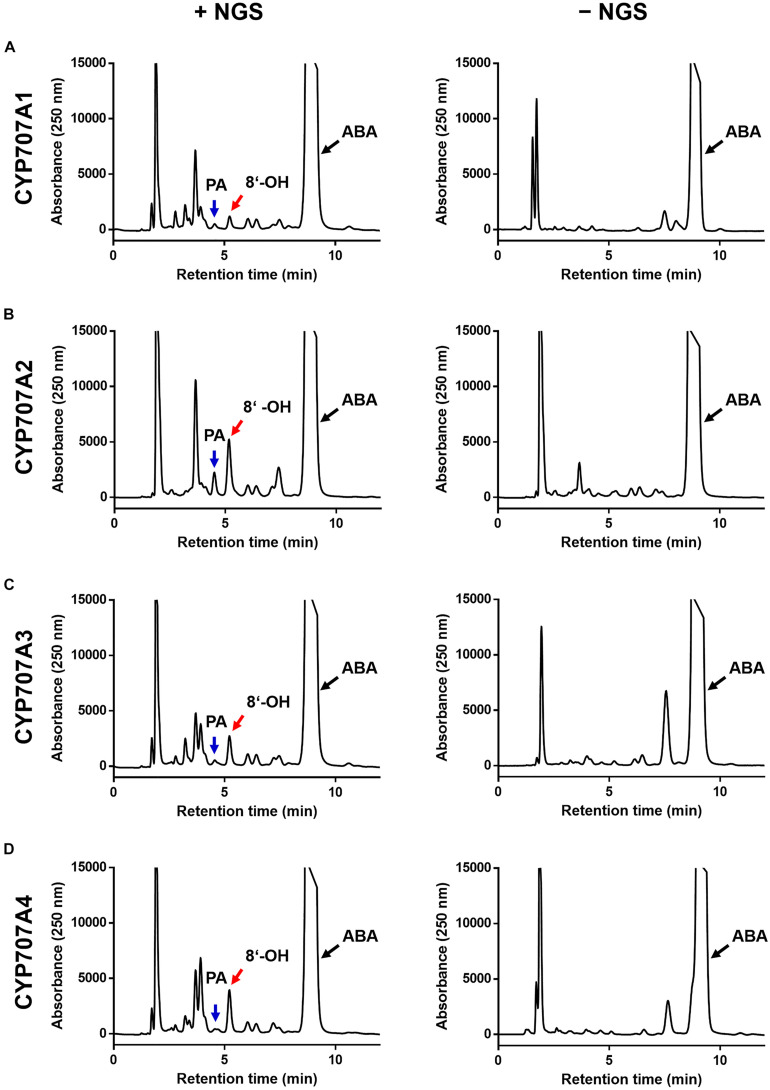
ABA hydroxylation activity of CaCYP707As. Characterization of CaCYP707A1 **(A)**, CaCYP707A2 **(B)**, CaCYP707A3 **(C)**, and CaCYP707A4 **(D)** function in ABA hydroxylation. CYP707A1, 2, 3, and 4 proteins expressed in *E. coli* were incubated with ABA in the presence (left; +NGS) or absence (right; –NGS) of a NADPH-generating system, and the catabolites were analyzed by HPLC. The peaks marked by red and blue arrows are 8′-hydroxy ABA (8′-OH) and phaseic acid (PA), respectively, whereas the peaks marked by black arrows are abscisic acid (ABA). The experiments were performed three times with similar results.

We next investigated whether the CaCYP707As had ABA hydroxylation activity (Figure 2). Previous studies demonstrated that CYP707As hydroxylate ABA to 8′-hydroxy ABA only in the presence of NADPH. The 8′-hydroxy ABA is spontaneously isomerized into PA, so that ABA hydroxylation by CYP707As results in production of 8′-hydroxy ABA or PA (Saito et al., 2004). When the CaCYP707A1 protein was incubated with ABA in the presence of NAPDH, 8′-hydroxy ABA and PA peaks were detected. By contrast, the peaks were not observed in NAPDH-free conditions in which CaCYP707As cannot act, indicating that CaCYP707A1 have the activity of ABA hydroxylation (Figure 2A). Similar to CaCYP707A1 proteins, CaCYP707A2, CaCYP707A3, and CaCYP707A4 proteins gave rise to 8′-hydroxy ABA and PA peaks only in the presence of NADPH, indicating that they also have ABA hydroxylation activity (Figures 2B–D). These findings indicate that the *CaCYP707A* genes encode CYPs responsible for ABA hydroxylation in *C. annuum*, and suggest that *CaCYP707A* is involved in ABA metabolism. This idea is partially supported by the result that transcriptional expression of *CaCYP707A* genes is regulated by abiotic stress such as dehydration conditions (Supplementary Figure 2).

### *CaCYP707A* Negatively Regulate Seed Formation

To understand the function of *CaCYP707A* genes in plant development, we generated transgenic tobacco plants overexpressing *CaCYP707A1* (*35S:CaCYP707A1*), *CaCYP707A2* (*35S:CaCYP707A2*), *CaCYP707A3* (*35S:CaCYP707A3*), and *CaCYP707A4* (*35S:CaCYP707A4*). In the *35S:CaCYP707A* transgenic plants, the ABA concentration was approximately 2–6 fold lower than that of NT grown in the same conditions for 6 weeks (Supplementary Figure 3). In addition, the transgenic plants exhibited a dehydration-like phenotype under normal growth conditions, unlike NT plants (Supplementary Figure 4). Because overexpression of *CaCYP707As* reduces ABA levels by promoting hydroxylation of ABA, a key hormone of stomatal closure, it was suggested that the dehydration-like phenotype of *CaCYP707As*-overexpressing plants might be caused by preventing ABA-induced stomatal closure.

**FIGURE 3:**
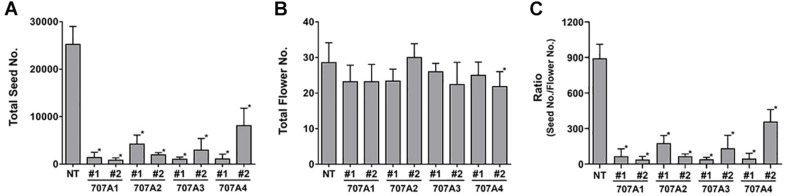
Overexpression of *CaCYP707As* suppresses seed formation. **(A)** Quantification of total seed number harvested from NT and *35S:CaCYP707A* plants grown in the same conditions (*n* > 5). 707A1, 2, 3, and 4 indicate *35S:CaCYP707A1*, *2*, *3*, and *4* transgenic plants, respectively. #1 and #2 are two individual lines of the indicated plants. **(B)** Quantification of total numbers of flowers formed in NT and *35S:CaCYP707As* plants (*n* > 5). **(C)** Ratio of total seed number to the number of flowers. Error bars are S.D. Asterisks indicate significant differences between the transgenic plants and NT controls (Student *t*-test *p* < 0.01). The experiments were performed three times with similar results.

**FIGURE 4:**
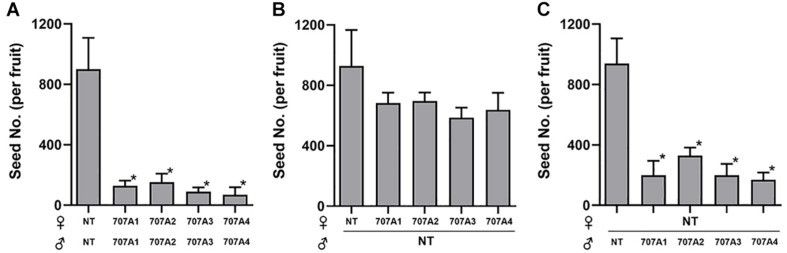
Cross-pollination rescues defective seed formation in *CaCYP707A*-overexpressing plants. **(A)** The number of seeds were quantified in the NT and transgenic fruits generated by self-pollination (*n* > 5). **(B)** The number of seeds were quantified in the *35S:CaCYP707As* transgenic fruits generated by cross-pollination using NT pollens (*n* > 5). **(C)** The number of seeds were quantified in the NT fruits generated by cross-pollination using the pollens of *35S:CaCYP707As* transgenic plants (*n* > 15). NT, 707A1, 2, 3, and 4 indicate non-transgenic, *35S:CaCYP707A1, 2, 3*, and *4*, respectively. Error bars are S.D. Asterisks indicate significant differences between NT control and the indicated samples (Student *t*-test, *p* < 0.01). Experiment **(A,B)** were performed three times with similar results.

Importantly, we observed that *35S:CaCYP707A* plants showed defects in seed formation, although there was no obvious difference in floral structure between NT and *35S:CaCYP707A* plants. The total number of seeds harvested from NT plants was approximately 25,000. However, total numbers of seeds collected from transgenic plants grown in the same conditions were much lower; *35S:CaCYP707A1* plants produced ∼800–1,400, *35S:CaCYP707A2* plants produced 2,000–4,200, *35S:CaCYP707A3* plants produced 1,000–3,000, and *35S:CaCYP707A4* plants produced 1,000–8,000 (Figure 3). The transgenic plants had produced similar or slightly lower numbers of flowers compared to wild-type plants. Consequently, the ratios of seed number and flower number in the transgenic plants were low compared to that of NT plants. This result also implies that the lower number of seeds in the transgenic plants is caused by defects in fertilization but not by defects in flower development. To explore this, we performed cross-pollination test. Seed formation in *CaCYP707As*-overexpressing plants was increased by cross-pollination using NT pollens. By contrast, seed formation in NT plants was decreased by cross-pollination using the pollens of *CaCYP707As*-overexpressing plants (Figure 4). This finding indicated that the suppressed seed formation in *35S:CaCYP707A* plants is caused by pollen defects in fertilization, suggesting that *CaCYP707A* are possibly involved in pollen development.

### *CaCYP707A* Genes Regulate Pollen Maturation

**FIGURE 5:**
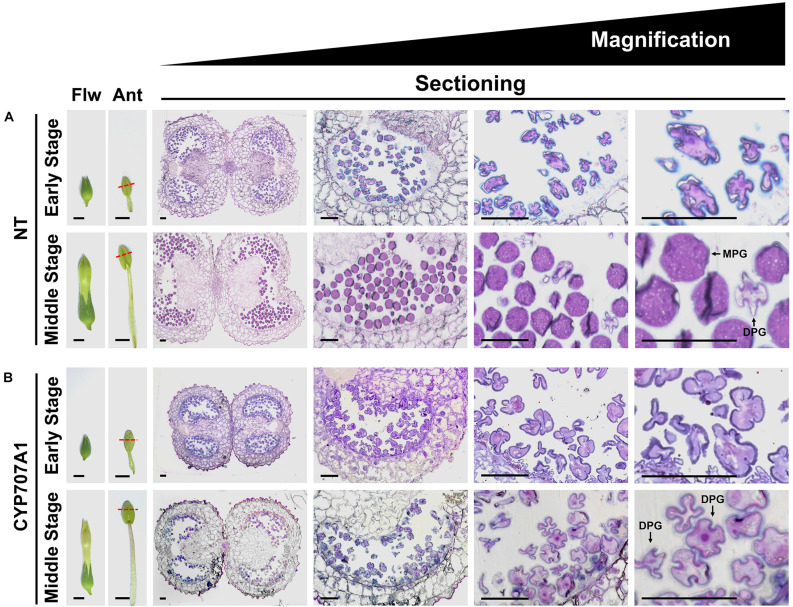
Overexpression of *CaCYP707A1* affects pollen maturation. **(Left)** Images of flowers (Flw) and anthers (Ant), and **(right)** semi-thin section images showing pollen development in early stage and middle-stage flowers of NT **(A)** and *35S:CaCYP707A1 #1* (CYP707A1) plants **(B)** grown in the same conditions. The slices were stained with toluidine blue O, and the images were captured by light microscope. Red dashed lines marked the longitudinal positions from which the slices were obtained. Ant, anther; DPG, degenerated pollen grain; Flw, flower; MPG, mature pollen grain. Scale bars = 0.5 cm in flowers, 0.2 cm in anthers, and 50 μm in sectioning images. More than three individuals of the indicated plants were analyzed, and the experiments were performed two times with similar results.

To understand the role of *CaCYP707A* genes in pollen development, NT and *35S:CaCYP707A1* anthers were collected from flowers at two different stages, and the morphology of the pollen was visualized by semi-thin sectioning and toluidine blue staining (Figure 5). In the early stage NT flowers, all pollen grains displayed a shrunken shape, and the phenotype was almost identical to that of *35S:CaCYP707A1* pollen grains. However, the morphologies of NT and *35S:CaCYP707A1* pollen in middle-stage flowers differed. In the middle-stage NT flowers, most pollen grains were mature pollen grains (MPGs) with a spherical shape and only a few pollen grains were degenerated pollen grains (DPGs) with a shrunken shape (Xu et al., 2017; Zhu et al., 2017). By contrast, in middle-stage *35S:CaCYP707A1* flowers, most pollen grains were DPGs, and a few pollen grains showed an intermediate phenotype between those of DPGs and MPGs. These findings indicate that *35S:CaCYP707A1* transgenic plants have defects in pollen maturation, suggesting that overexpression of *CaCYP707A1* negatively regulates maturation of pollen.

**FIGURE 6:**
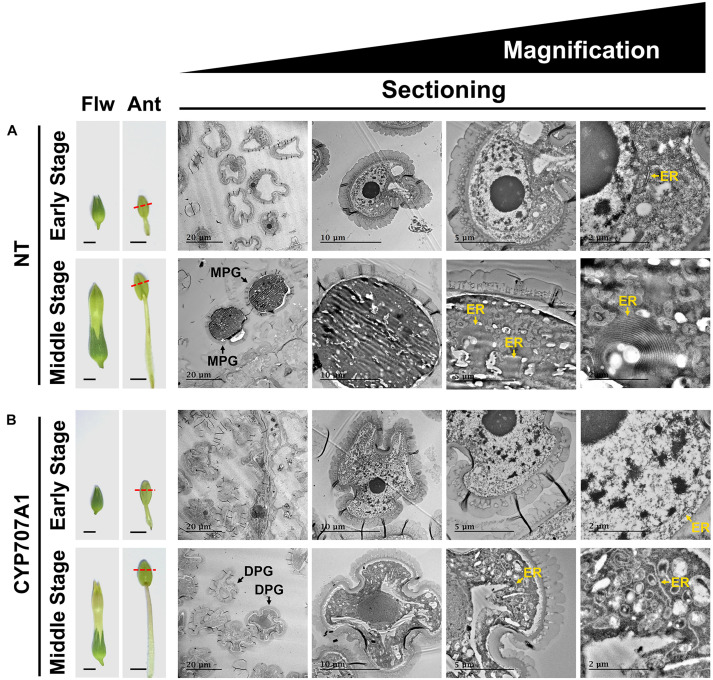
Suppressed pollen maturation in *35S:CaCYP707A1* plants. **(Left)** Images of flowers (Flw) and anthers (Ant), and **(right)** ultrastructure of the pollen formed in NT **(A)** and *35S:CaCYP707A1* **(B)** plants as shown by ultra-thin sections and TEM. CYP707A1 indicates *35S:CaCYP707A1* transgenic plants (line #1), and red dashed lines mark the longitudinal positions from which the slices were obtained. Yellow arrows indicate the ER membrane. Ant, anther; DPG, degenerated pollen grain; ER, endoplasmic reticulum; Flw, flower; MPG, mature pollen grain. Scale bars = 0.5 cm in flower; 0.2 cm in anther; and (left to right) 20, 10, 5, and 2 μm in TEM images. Two individuals of the indicated plants were analyzed, and the experiments were performed two times with similar results.

To further explore their maturation, we examined the ultrastructure of *35S:CaCYP707A1* pollen by TEM (Figure 6). Similar to the results from light microscopy, we observed morphological differences between NT and *35S:CaCYP707A1* pollen only in the middle-stage flowers with mature pollen grains, but not in early stage flowers with immature pollen grains. Notably, TEM analysis showed that ER development, which is tightly linked to pollen maturation (Kim et al., 2011), was different between NT and *35S:CaCYP707A1* pollen in middle-stage flowers. In middle-stage flowers, we observed well-developed ER only in NT pollen, but not in *35S:CaCYP707A1* pollen. However, this difference in ER formation was not observed between NT and *35S:CaCYP707A1* pollen in early stage flowers (Figure 6). These findings support the conclusion that overexpression of *CaCYP707A1* suppresses pollen maturation.

### *CaCYP707As* Negatively Regulate Pollen Viability

**FIGURE 7:**
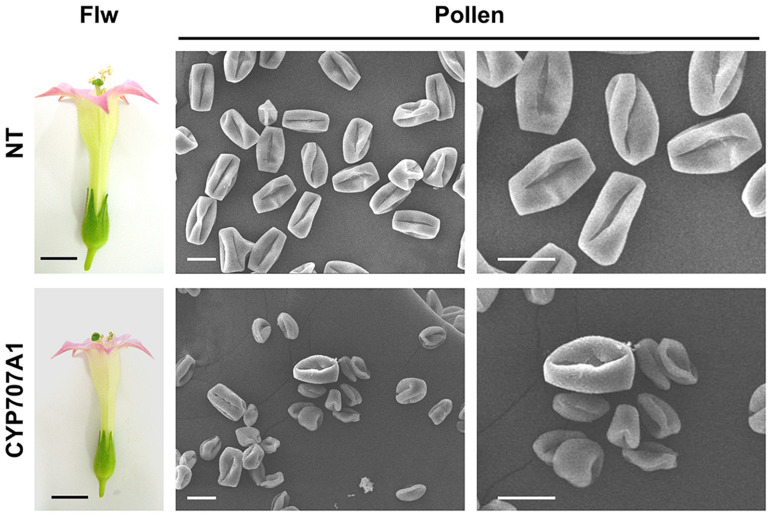
Overexpression of *CaCYP707A1* promotes formation of immature pollen. Pollen maturation was investigated by SEM. Pollen of NT and *35S:CaCYP707A1* (line # 1) were collected from the anthesis stage of flowers **(left)**, and morphological patterns of the pollen were captured by SEM **(right)**. Flw and CYP707A1 indicate flowers and *35S:CaCYP707A1* plants, respectively. Scale bars = 1 cm in flowers and 20 μm in SEM images. Two individuals of the indicated plants were analyzed, and the experiments were performed two times with similar results.

To further test the hypothesis that *CaCYP707A1* negatively regulates maturation of pollen, we analyzed the morphologies of the NT and *CaCYP707A1*-overexpressing plant pollen at the anthesis stage using SEM. In NT plants, most pollen grains had similar size and shape. The *35S:CaCYP707A1* flowers formed a few pollen grains whose phenotype (size and shape) was similar to that of the NT pollen; however, most pollen in the transgenic flowers was smaller and more irregular compared to the pollen of NT flowers (Figure 7). This SEM result supports the TEM result showing that maturation of pollen is defective in *35S:CaCYP707A1* plants. In addition, the result showing that ABA concentration in the anthers of *35S:CaCYP707A1* plants is much lower than that of NT plants (Supplementary Figure 5) suggests that *CaCYP707A*-promoted ABA hydroxylation inhibits pollen maturation. Similar to *CaCYP707A1*, overexpression of *CaCYP707A2, CaCYP707A3* or *CaCYP707A4* also suppressed pollen maturation (Supplementary Figure 6), further supporting the function of *CaCYP707As* in pollen development.

**FIGURE 8:**
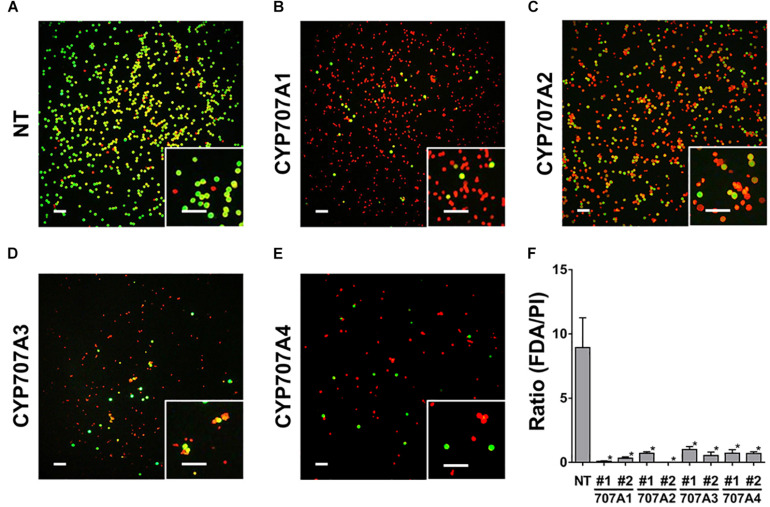
Suppressed pollen viability in *35S:CaCYP707A* plants. Pollen viability was visualized by FDA and PI staining. Pollen collected from the flowers (anthesis stage) of NT and *35S:CaCYP707A* transgenic plants were stained with a mixture of FDA and PI for 5 min, and then fluorescent signals were observed by fluorescence microscopy [**(A)**, NT; **(B)**, CaCYP707A1 #1; **(C)**, CaCYP707A2 #1; **(D)**, CaCYP707A3 #1; and **(E)**, CaCYP707A4 #1]. More than three individuals of the indicated plants were analyzed with similar results. **(F)** Quantification of the ratio of FDA (green) to PI (red) signals in the indicated plants (*n* > 3). The values are the means of three biological replicates, and error bars are S.D. #1 and #2 indicate individual lines of the indicated transgenic plants. Asterisks indicate significant differences between the transgenic plants and NT controls (Student *t*-test *p* < 0.01). Scale bars = 100 μm. The experiments were performed at least three times with similar results.

To address how the defect in pollen maturation leads to the inhibited seed formation in *35S:CaCYP707As* plants, we tested pollen viability in NT and *35S:CaCYP707A1* plants. To do this, we stained pollens at the anthesis stage with FDA and PI, and monitored the fluorescent signals of FDA and PI to visualize viability (Figure 8). In this assay, viable pollen exhibit green fluorescence signals upon FDA staining, whereas non-viable pollen display red fluorescence signals upon PI staining. In NT plants, most pollen displayed green fluorescence signals upon FDA staining. In contrast to the NT plants, most pollen from *35S:CaCYP707A1* plants showed red fluorescence signals upon PI staining, whereas only a small portion of the pollen exhibited green fluorescence signals (Figures 8A,B). We obtained nearly identical results for the pollen viability of plants expressing CaCYP707A2, 3, and 4. Most pollen in *35S:CaCYP707A2*, *3*, and *4* plants displayed red signals corresponding to PI, and only a few pollen exhibited green signals corresponding to FDA (Figures 8C–E). When we determined the ratio of the number of pollen with FDA signals to the number of pollen with PI signals, there was an obvious difference in pollen viability (Figure 8F). The ratios of the transgenic plants were much lower than those of the NT controls. This finding indicates that *CaCYP707A*-promoted ABA hydroxylation negatively regulate pollen viability, explaining why seed formation was inhibited in *35S:CaCYP707A* plants.

## Discussion

The plant CYPs are the third largest gene family in higher plants (Nelson and Werck-Reichhart, 2011). Unlike bacterial CYPs, most plant CYPs with transmembrane domains are mainly located at the ER, and some are predicted to be located at the membranes of other organelles such as PM and plastids (Schuler et al., 2006; Bak et al., 2011; Yin-Ping et al., 2019). CYPs act as monooxygenases, and their activity is regulated by NADPH and NADPH-cytochrome P450 reductase (CPR), which provide electrons to CYPs (Jensen and Moller, 2010; Bak et al., 2011). In plants, CYPs are involved in plant growth and physiology, mediating a variety of metabolic processes, including metabolism of hormones such as auxin, cytokinin, and ABA (Kim and Tsukaya, 2002; Mizutani, 2012). In this study, we identified four CYP707As (CaCYP707A1, 2, 3, and 4) in *C. annuum* and characterized their function in ABA degradation. Production of 8′-hydroxy ABA is a key step in ABA degradation (Saito et al., 2004), and our enzymatic assay showed that the CaCYP707As mediate production of 8′-hydroxy ABA from ABA only in the presence of NADPH. In addition, overexpression of *CaCYP707A* genes reduced ABA levels and induced a dehydration phenotype, indicating that the CaCYP707As mediate ABA degradation by controlling hydroxylation of ABA.

In this study, we observed that seed formation is suppressed by overexpression of *CaCYP707A* genes. The total numbers of seeds formed in *35S:CaCYP707A1* plants were ∼20-fold lower than those of NT plants, and the defective phenotype was also observed in other transgenic plants overexpressing *CaCYP707A2*, *3*, and *4*. This indicates that CaCYP707A1-mediated ABA degradation regulates seed formation. Previous studies using ABA biosynthesis mutants support this finding. ABA-deficient mutant *aba2-1* shows suppressed ABA response and forms fewer seeds per silique in Arabidopsis (Sharma and Verslues, 2010; Cheng et al., 2014), and a knockdown mutant of *NCED*, which is responsible for ABA biosynthesis, also exhibits lower levels of ABA than NT control plants, leading to fewer seeds per fruit (Sun et al., 2012). Together, these findings suggest that CaCYP707A-mediated degradation of ABA induces defects in seed formation, and the cross-pollination result showing that NT pollen rescued the defective seed phenotype in *35S:CaCYP707A* plants further indicates that the defective seed phenotype is due to defects in pollen development in *CaCYP707A*-overexpressing plants.

A recent study by Dai et al. (2018) using *NCED* suggested that ABA is involved in pollen development in tomato. In that study, the authors showed that *RNAi* gene silencing of *NCED* reduced the ABA levels in the *RNAi* transgenic tomato and suppressed pollen development. Interestingly, overexpression of *NCED* also suppressed pollen development, although the overexpression increased ABA levels, suggesting that pollen development is sensitive to ABA homeostasis. A study by Saini and Aspinall (1982) showing that exogenous ABA affects pollen development partially supports this hypothesis. In our study, we traced pollen development with age, and found that maturation of immature pollen grains is suppressed in *35S:CaCYP707A1* transgenic plants. This indicated that overexpression of *CaCYP707A* genes suppresses pollen maturation, and ultra-thin sectioning and TEM results visualizing ER formation further supported this finding. Establishment of the intracellular membrane network during pollen maturation is characterized by ER development (Rodríguez-García et al., 1995; Piffanelli et al., 1998). Therefore, the TEM results that middle-stage *35S:CaCYP707A1* pollen had a poorly developed ER unlike the same developmental stage of NT pollens with a well-developed ER support the hypothesis that *CaCYP707A* negatively regulates pollen maturation. In addition, our SEM and pollen viability results show that the defect in pollen maturation affects pollen development and pollen viability. NT pollen grains at the anthesis stage had similar size and shape, and also were viable, whereas pollen grains of *35S:CaCYP707A1* plants were irregular in shape and non-viable. Additionally, in response to exogenous ABA treatment, pollen maturation in NT and *CaCYP707A1*-overexpressing plants tended to be reduced and enhanced, respectively (Supplementary Figure 7). This indicates that overexpression of *CaCYP707A1* suppressed by promoting ABA hydroxylation, and also suggests that ABA homeostasis is involved in pollen maturation. This finding is partially supported by a previous study by Dai et al. (2018), which shows that ABA homeostasis affects pollen development (Dai et al., 2018). Together with the results showing that *CaCYP707A1* overexpression reduced ABA levels in anthers, these findings suggest that overexpression of *CaCYP707As* suppresses pollen maturation and seed formation by promoting ABA hydroxylation.

## Data Availability Statement

The datasets presented in this study can be found in online repositories. The names of the repository/repositories and accession number(s) can be found in the article/Supplementary Material.

## Author Contributions

HMK and YHJ planned and designed all aspects of the research. HMK and CHY analyzed the data and provided technical guidance in enzyme assay experiments. HMK and SHP generated plant gene expression constructs. HMK, SHM, and SYP participated phenotypical analysis of plant. HMK, GJ, and YHJ performed the data interpretation and wrote the manuscript. All authors contributed to the article and approved the submitted version.

## Conflict of Interest

The authors declare that the research was conducted in the absence of any commercial or financial relationships that could be construed as a potential conflict of interest.

## Supplementary Material

Supplementary Figure 1 Multiple alignment of CaCYP707As amino acid sequence.

Supplementary Figure 2 *CaCYP707As* expression in response to drought.

Supplementary Figure 3 ABA concentration of *CaCYP707As* transgenic plant leaves.

Supplementary Figure 4 Dehydration phenotype of *CaCYP707As* transgenic plants.

Supplementary Figure 5 ABA concentration of *CaCYP707A1* transgenic plant anther.

Supplementary Figure 6 Morphological patterns of *CaCYP707A2, 3*, and *4* transgenic plant pollen.

Supplementary Figure 7 Pollen maturation of *CaCYP707A1* transgenic plant under ABA treatment.

Supplementary Table 1 A list of primer sequences used in this study.
