# Supplementary material for: Promoted ABA Hydroxylation by Capsicum annuum CYP707As Overexpression Suppresses Pollen Maturation in Nicotiana tabacum
Source: Front Plant Sci. 2020 Dec 8;11:583767. doi: 10.3389/fpls.2020.583767 (PMC7752897; doi:10.3389/fpls.2020.583767)
Supplement: Supplementary Figure 1 — Multiple alignment of CaCYP707As amino acid sequence. [file Data_Sheet_1.docx]

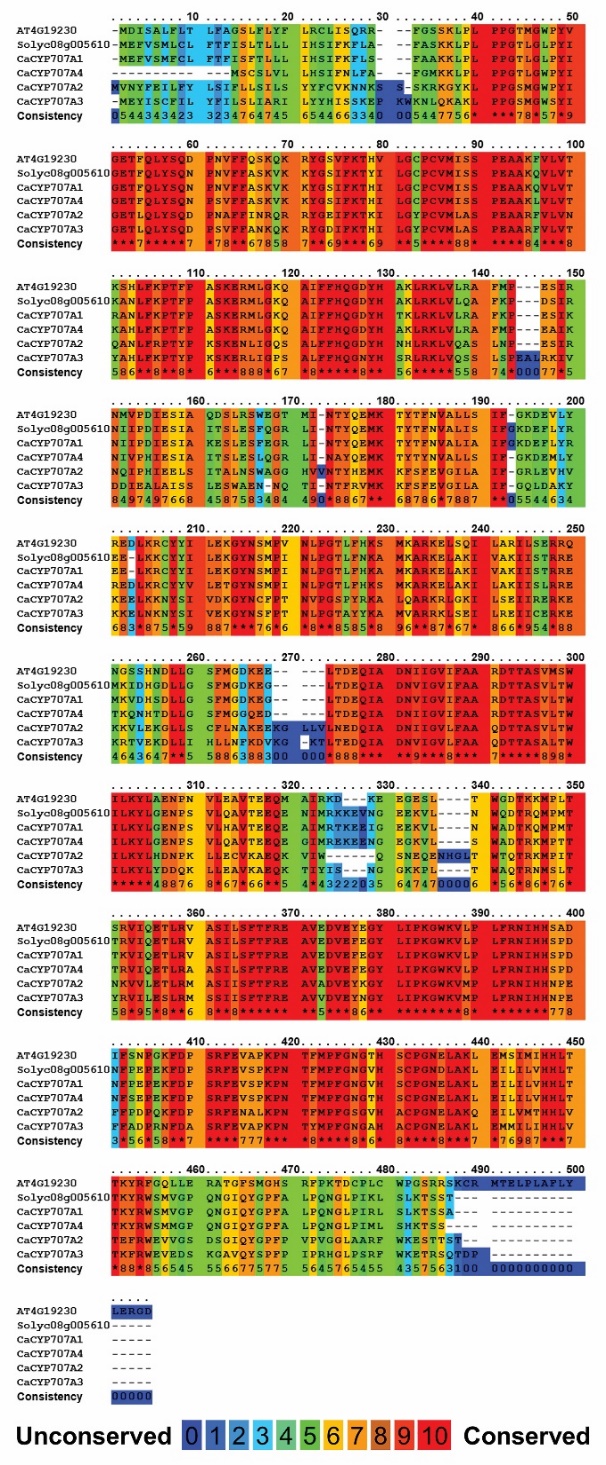


**Supplementary Figure S1. CaCYP707As are conserved with CYP707As of Arabidopsis and tomato.**

To investigate conservation of CaCYP707As with CYP707As of Arabidopsis and tomato, multiple sequence alignments were performed by the PRALINE program (http://www.ibi.vu.nl/programs/pralinewww/) using full-length amino acid sequences of the indicated CYP707As. Darker red boxes indicate a higher level of conservation, whereas darker blue boxes indicate a lower level of conservation.


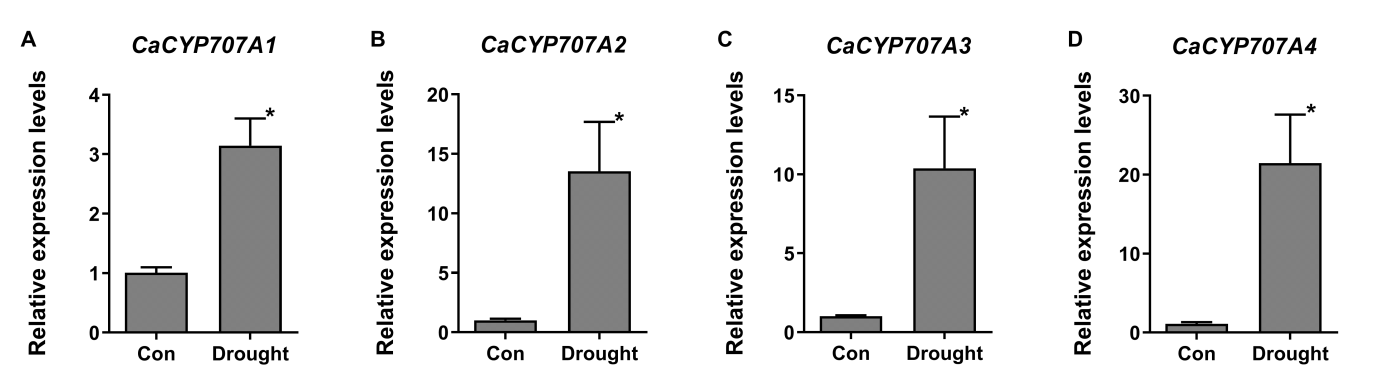


**Supplementary Figure S2. Drought upregulates expression of *CaCYP707A* genes**

Regulation of *CaCYP707A* geneexpression by drought was analyzed by PCR. Total RNA was extracted from leaves drought-untreated (Con) or drought-treated for 6 h. *CaACT* (DQ832719), *CaUBI-3* (DQ924970), and *Caβ-TUB* (EF495259) were used as reference genes to normalize expression of *CaCYP707A* genes. The values are the means of three biological replicates with three technical replicates, and error bars are S.D.Asterisks indicate significant differences between drought-treated samples and untreated controls (Student t-test *p*< 0.01).

**
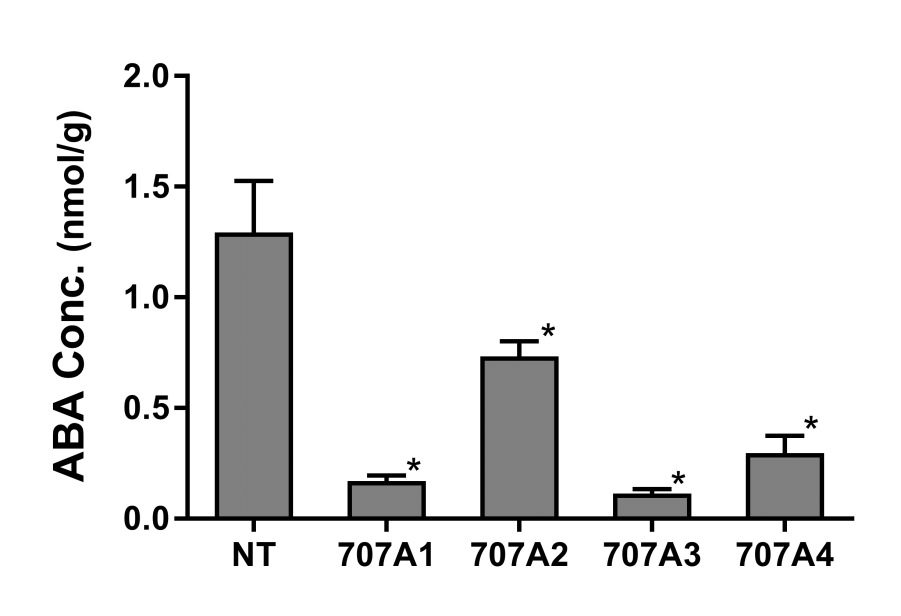
**

**Supplementary Figure S3. Reduced concentration of ABA in *35S::CYP707A* plant leaves.**

ABA concentration was quantified in 6-week-old NT and *35S::CYP707As* transgenic plants grown in the same conditions. 707A1, 2, 3, and 4 indicate *35S::CYP707A*1, 2, 3, and 4 transgenic plants.The values are the means of two biological replicates with three technical replicates, and error bars are S.D. Asterisks indicate significant differences between the transgenic plants and NT controls (Student t-test *p*< 0.03).


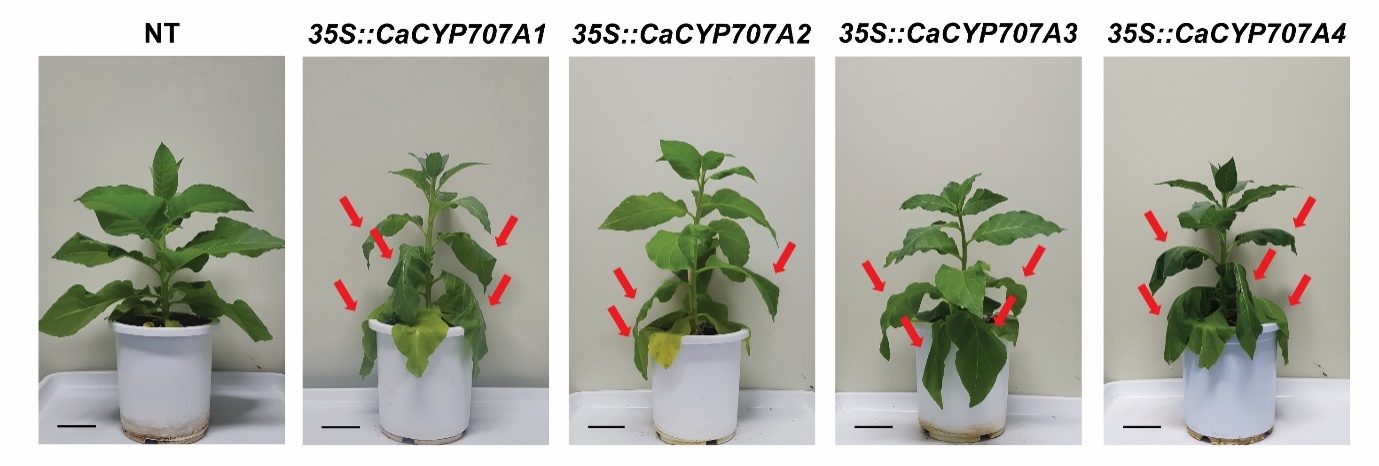


**Supplementary Figure S4. Overexpression of *CaCYP707A* genes induces a dehydration phenotype.**

Phenotypes of NT and *35S::CaCYP707A* transgenic plants grown in the same conditions for 6 weeks are shown. Red arrows indicate leaves showing the dehydration phenotype. Scale bars = 5 cm. The experiments were performed at least three times with similar results.


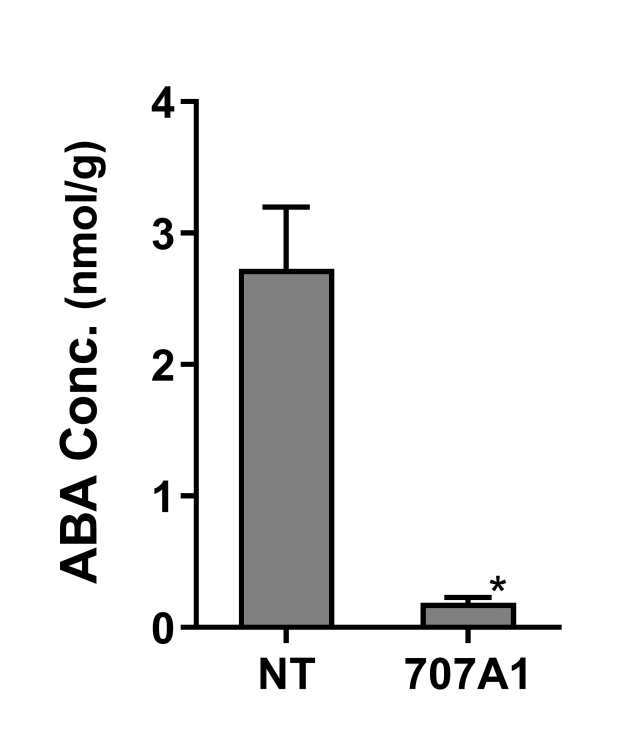


**Supplementary Figure S5. Overexpression of CaCYP707A1 suppresses accumulation of ABA in anthers.**

ABA concentration was quantified in flowers at the anthesis stage. 707A1 indicates *35S::CYP707A1*plants.The values are the means of two biological replicates with three technical replicates, and error bars are S.D. Asterisk indicates significant difference between the *35S::CYP707A1*transgenic plants and NT controls (Student t-test *p*< 0.01).

**
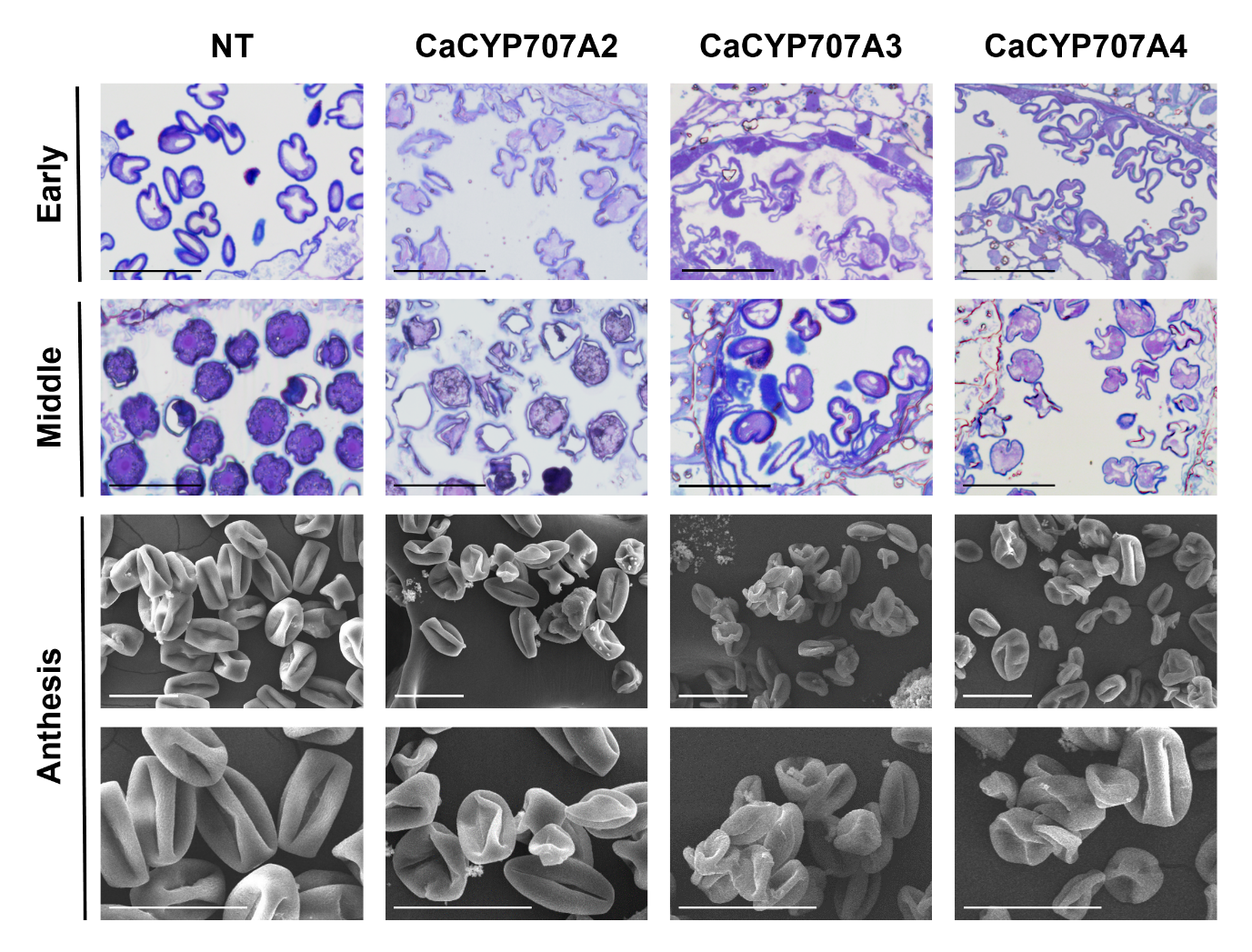
**

**Supplementary Figure S6. Overexpression of *CaCYP707A2, 3* and *4* affects pollen maturation**

Pollen development in *CYP707A2, 3,* and *4*-overexpressingtransgenic plants was analyzed by light and scanning electron microscope. The pollens in early and middle stage of flowers were visualized by semi-thin sectioning and light microscope, and the pollens in anthesis stage of flowers were visualized by scanning electron microscope. CaCYP707A2, 3 and 4 indicate the *35S::CYP707A2, 3* and *4* plants, respectively. Scale bars = 50 μm. More than three individuals of the indicated plants were analyzed with similar results.


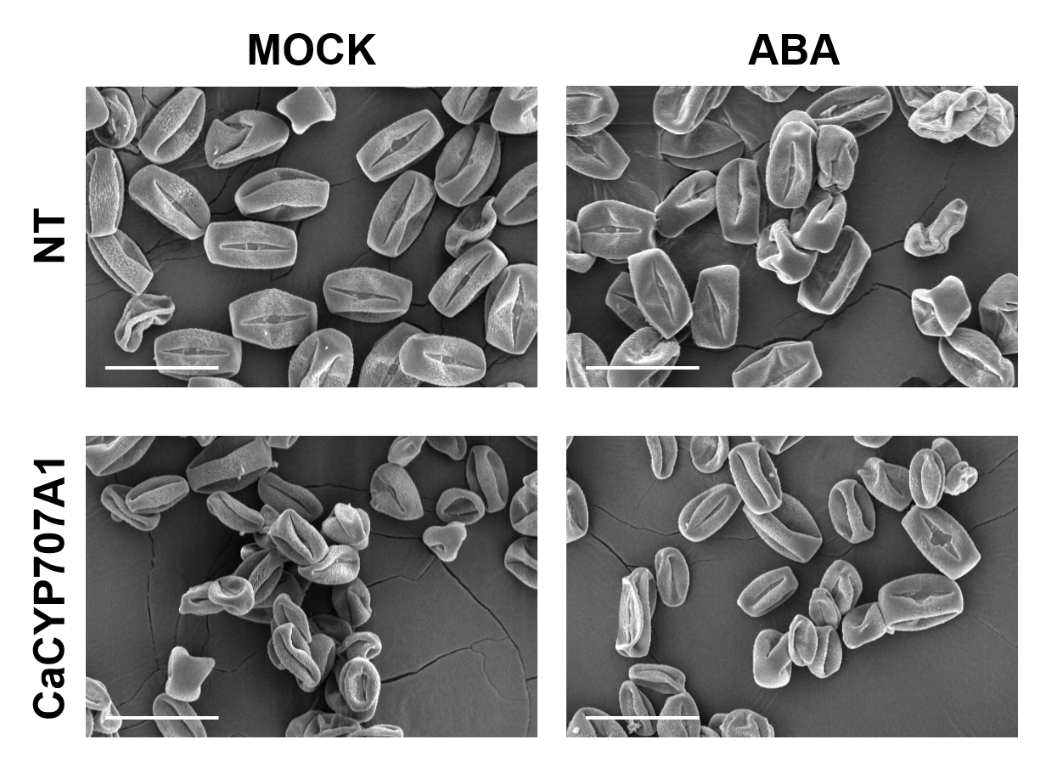


**Supplementary Figure S7. ABA partially rescued the defective pollen maturation in *35S::CaCYP707A1* plants.**

To analyze the effect on exogenous ABA to pollen development, early stage of flowers of NT and *35S::CaCYP707A1* transgenic plants were treated with Mock (0.1% Tween-20) and 100 μM ABA by spaying every two days. The pollens collected from the flowers at anthesis stage were visualized by SEM. CaCYP707A1 indicates the *35S::CYP707A1* plant. Scale bars = 50 μm. Three individuals of the indicated plants were tested with similar results.

**Table S1.** List of primers used in this study.

| Primer Name |  | Primer Sequence (5'-3') | restriction enzyme | Purpose |
| --- | --- | --- | --- | --- |
| CaCYP707A1-ORF-F |  | ATGGAATTTGTTTCTATGTTTTGTTTG |  | Gene isolation |
| CaCYP707A1-ORF-R |  | TTATGCTGATGATGTCTTAAGAGA |  | Gene isolation |
| CaCYP707A2-ORF-F |  | ATGGTTAATTATTTTGAAATATTATTCTA |  | Gene isolation |
| CaCYP707A2-ORF-R |  | TTAGGTTGATGTAGTAGATTCTTTC |  | Gene isolation |
| CaCYP707A3-ORF-F |  | ATGGAGTATATTTCTTGTTTCATTCTT |  | Gene isolation |
| CaCYP707A3-ORF-R |  | CTAGGGATCTGTTTGGCTTC |  | Gene isolation |
| CaCYP707A4-ORF-F |  | ATGTCTTGTTCCCTTGTACTGAG |  | Gene isolation |
| CaCYP707A4-ORF-R |  | TTATGATGAAGTCTTGTGGGAGAG |  | Gene isolation |
| KpnⅠ-EGFP-F |  | GGTACCATGGTGAGCAAGG | KpnⅠ | GFP fusion cloning |
| SacⅠ-EGFP-R |  | GAGCTCTCAGTGGTGGTG | SacⅠ | GFP fusion cloning |
| CaCYP707A1-GFP-F |  | GGTACCATGGAATTTGTTTCTATG | KpnⅠ | GFP fusion cloning |
| CaCYP707A1-GFP-R |  | GGTACCTGCTGATGATGTCTT | KpnⅠ | GFP fusion cloning |
| CaCYP707A2-GFP-F |  | GACTCTAGTGGATCCATGGTT | BamHⅠ | GFP fusion cloning |
| CaCYP707A2-GFP-R |  | GGTACCGGTTGATGTAGTAG | KpnⅠ | GFP fusion cloning |
| CaCYP707A3-GFP-F |  | GGTACCATGGAGTATATTTCTTG | KpnⅠ | GFP fusion cloning |
| CaCYP707A3-GFP-R |  | GGTACCGGGATCTGTTTGG | KpnⅠ | GFP fusion cloning |
| CaCYP707A4-GFP-F |  | GGTACCATGTCTTGTTCCCTTG | KpnⅠ | GFP fusion cloning |
| CaCYP707A4-GFP-R |  | GGTACCTGATGAAGTCTTGTG | KpnⅠ | GFP fusion cloning |
| CaCYP707A1-SacⅠ-F |  | GAGCTCATGGAATTTGTTTCTATGT | SacⅠ | pCW, pBI121 cloning |
| CaCYP707A1-SacⅠ-R |  | GAGCTCTTATGCTGATGATGTCT | SacⅠ | pCW, pBI121 cloning |
| CaCYP707A2-BamHⅠ-F |  | CAAAGGATCCATGGTTAATTATTTTGA | BamHⅠ | pCW, pBI121 cloning |
| CaCYP707A2-BamHⅠ-R |  | AGAAGGATCCTTAGGTTGATGTAGTAG | BamHⅠ | pCW, pBI121 cloning |
| CaCYP707A3-BamHⅠ-F |  | GGATCCATGGAGTATATTTCTTGTTTCA | BamHⅠ | pCW, pBI121 cloning |
| CaCYP707A3-SacⅠ-R |  | GAGCTCCTAGGGATCTGTTTGG | SacⅠ | pCW, pBI121 cloning |
| CaCYP707A4-SacⅠ-F |  | GAGCTCATGTCTTGTTCCCTTGTACTA | SacⅠ | pCW, pBI121 cloning |
| CaCYP707A4-SacⅠ-R |  | GAGCTCTTATGATGAAGTCTTGTGG | SacⅠ | pCW, pBI121 cloning |
| CaACT-F |  | CGGAATCCACGAGACTACAT |  | RT-qPCR |
| CaACT-R |  | GGGAAGCCAAGATAGAGCCT |  | RT-qPCR |
| CaUBI-3-F |  | TGTCCATCTGCTCTCTGTTG |  | RT-qPCR |
| CaUBI-3-R |  | CACCCCAAGCACAATAAGAC |  | RT-qPCR |
| CaB-TUB-F |  | GAGGGTGAGTGAGCAGTTC |  | RT-qPCR |
| CaB-TUB-R |  | CTTCATCGTCATCTGCTGTC |  | RT-qPCR |
| CaCYP707A1-RT-F |  | CACATTTAGAGAAGCTGTTGAAG |  | RT-qPCR |
| CaCYP707A1-3'-R |  | GTGATTTTCTTGATTGGCTTATGC |  | RT-qPCR |
| CaCYP707A2-RT-F |  | GAGTGCACGCTTGTCCAG |  | RT-qPCR |
| CaCYP707A2-3'-R |  | GCAAATCAGAAGAGCCATTAGG |  | RT-qPCR |
| CaCYP707A3-RT-F |  | CTAAAGGTTGGAAGGTGATGC |  | RT-qPCR |
| CaCYP707A3-3'-R |  | ATATCAGGAGGCATCTCGTTT |  | RT-qPCR |
| CaCYP707A4-RT-F |  | CAGAGAAGCTGTTGAAGATGTTG |  | RT-qPCR |
| CaCYP707A4-3'-R |  | ATTTTACTGTGTATTTATGATGAAGTCTTG |  | RT-qPCR |

* The restriction enzyme site is underlined.
